# Cre Recombinase Driver Mice Reveal Lineage-Dependent and -Independent Expression of Brs3 in the Mouse Brain

**DOI:** 10.1523/ENEURO.0252-21.2021

**Published:** 2021-08-16

**Authors:** Allison S. Mogul, Colleen K. Hadley, Haley S. Province, Jordan Pauli, Oksana Gavrilova, Cuiying Xiao, Richard D. Palmiter, Ramón A. Piñol, Marc L. Reitman

**Affiliations:** 1 Diabetes, Endocrinology, and Obesity Branch, National Institute of Diabetes and Digestive and Kidney Diseases, National Institutes of Health, Bethesda, Maryland 20892; 2 Department of Biochemistry, Genome Sciences, and Howard Hughes Medical Institute, University of Washington, Seattle, Washington 98195; 3 Mouse Metabolism Core, National Institute of Diabetes and Digestive and Kidney Diseases, National Institutes of Health, Bethesda, Maryland 20892

**Keywords:** bombesin receptor subtype-3, BRS3, Cre recombinase driver mice, dentate gyrus, hypothalamus, lineage tracing, parabrachial nucleus

## Abstract

Bombesin receptor subtype-3 (BRS3) is an orphan receptor that regulates energy homeostasis. We compared *Brs3* driver mice with constitutive or inducible Cre recombinase activity. The constitutive BRS3-Cre mice show a reporter signal (Cre-dependent tdTomato) in the adult brain because of lineage tracing in the dentate gyrus, striatal patches, and indusium griseum, in addition to sites previously identified in the inducible BRS3-Cre mice (including hypothalamic and amygdala subregions, and parabrachial nucleus). We detected *Brs3* reporter expression in the dentate gyrus at day 23 but not at postnatal day 1 or 5 months of age. Hypothalamic sites expressed reporter at all three time points, and striatal patches expressed *Brs3* reporter at 1 day but not 5 months. Parabrachial nucleus *Brs3* neurons project to the preoptic area, hypothalamus, amygdala, and thalamus. Both Cre recombinase insertions reduced *Brs3* mRNA levels and BRS3 function, causing obesity phenotypes of different severity. These results demonstrate that driver mice should be characterized phenotypically and illustrate the need for knock-in strategies with less effect on the endogenous gene.

## Significance Statement

Bombesin receptor subtype-3 (BRS3) expression is a marker for selected neurons that regulate body temperature and energy metabolism, among other functions. BRS3-Cre recombinase driver mice allow investigation of these neurons, demonstrating discrete populations with stable (including hypothalamic and amygdala subregions, parabrachial nucleus) and developmentally transient expression (dentate gyrus, striatal patches). These mice also illustrate the need for knock-in strategies having less effect on proper expression of the endogenous gene.

## Introduction

Bombesin receptor subtype-3 (BRS3, Bombesin-like receptor 3, BB3) is a G-protein-coupled receptor critical for the maintenance of energy balance (Jensen et al., 2008; González et al., 2015; Xiao and Reitman, 2016). BRS3 is considered an orphan receptor as its endogenous ligand is not known; specifically, it does not bind the natural ligands (gastrin-releasing peptide and neuromedin B) for the most closely related receptors (Mantey et al., 1997); nor does it bind bombesin, which is the frog ortholog of neuromedin B (Hirooka et al., 2021). Mice lacking BRS3 are hyperphagic, have a reduced resting metabolic rate and body temperature, and consequently become obese (Ohki-Hamazaki et al., 1997b). *Brs3* knock-out (KO) mice are also reported to exhibit reduced social responses, a heightened preference for sweetness, and increased aversion to bitterness (Yamada et al., 1999, 2000). Although *Brs3* is also expressed outside the CNS (Jensen et al., 2008), the metabolic phenotypes of *Brs3* KO mice are predominantly mediated by glutamatergic *Brs3* neurons, with contributions from neurons expressing MC4R and SIM1 demonstrating the necessity of brain BRS3 (Xiao et al., 2017, 2020). Consistent with the phenotypes of BRS3*-*null mice, administration of a BRS3 agonist reduces food intake and body weight and increases brown adipose tissue (BAT)-induced thermogenesis, heart rate, and blood pressure, while BRS3 antagonists increase food intake and body weight (Guan et al., 2010, 2011; Nio et al., 2017; Maruyama et al., 2018). Single doses of a BRS3 agonist increased blood pressure in humans (Reitman et al., 2012), likely via increased sympathetic tone (Lateef et al., 2016), reducing interest in central agonism as a human therapeutic for obesity and stimulating interest in studying BRS3 agonists that do not enter the brain (Kiyotsuka et al., 2016).

While pharmacologic and genetic manipulations of BRS3 have revealed the functions of the receptor, BRS3 can also be used as a marker for studying the neural circuitry regulating energy homeostasis. BRS3 is found in discrete brain regions in the mouse (Ohki-Hamazaki et al., 1997a; Yamada et al., 1999; Guan et al., 2010; Zhang et al., 2013; Piñol et al., 2018; see also *Allen Mouse Brain Atlas*) and other species (Liu et al., 2002; Sano et al., 2004; Zhang et al., 2013; Maruyama et al., 2018). The contributions of hypothalamic *Brs3* neurons to energy homeostasis are heterogeneous. *Brs3* neurons in the paraventricular hypothalamus (PVH) regulate food intake, but not body temperature or BAT thermogenesis. In contrast, *Brs3* neurons in the preoptic area (POA) of the hypothalamus and dorsomedial hypothalamus (DMH) regulate body temperature, energy expenditure, heart rate, and blood pressure, but not appetite (Piñol et al., 2018, 2021). POA *Brs3* neurons have also been implicated in parental behaviors (Moffitt et al., 2018; Yoshihara et al., 2021).

We previously generated a tamoxifen-dependent BRS3-Cre driver mouse line (hereafter called BRS3-CreER) and used it to elucidate the anatomy, connectivity, and physiology of neurons expressing *Brs3* (Piñol et al., 2018). Here we characterize a new, constitutive BRS3-Cre allele (hereafter called BRS3-IRES (internal ribosomal entry site)-Cre) and show that the BRS3-IRES-Cre driver can be used for lineage tracing. In both BRS3-Cre lines, the *Cre* insertion affects *Brs3* expression and function in whole-body physiology. These results provide guidance and have general implications for developing driver mice.

## Materials and Methods

### Mice

Procedures were approved by the National Institute of Diabetes and Digestive and Kidney Diseases and the University of Washington Animal Care and Use Committees (protocols K016-DEOB-20 and 2183–02, respectively). The following mice were used: C57BL/6J (catalog #000664, The Jackson Laboratory); Ai14, with Cre-dependent expression of tdTomato (catalog #007914, The Jackson Laboratory; Madisen et al., 2010); BRS3-CreER, with tamoxifen-dependent Cre activity (catalog #032614, The Jackson Laboratory; allele is *Brs3^tm3.1(cre/ERT2)Rei^*; Piñol et al., 2018); and BRS3-IRES-Cre, with constitutive Cre activity (described below; catalog #030540, The Jackson Laboratory; allele is *Brs3^tm1.1(cre/GFP)Rpa^*). BRS3-CreER;Ai14 mice showed no evidence of tamoxifen-independent germline recombinase activity in >100 progeny as assessed by the lack of generalized tdTomato expression. Mice were studied on a C57BL/6J background. Male mice were used since *Brs3* is on the X chromosome and undergoes X-inactivation (Piñol et al., 2018). Mice were housed on a 12 h dark/light cycle (lights on at 6:00 A.M.), at ∼22°C, with Teklad bedding (catalog #7090, Envigo) and *ad libitum* access to water and chow (15% kcal fat; energy density, 3.1 kcal/g; catalog #7022 NIH-07 diet; Envigo) or high fat diet (HFD; 60% kcal fat; 5.24 metabolizable kcal/g; catalog #D12492, Research Diets) in a clean conventional vivarium. BRS3-CreER mice were genotyped as described previously (Piñol et al., 2018). Both BRS3-CreER and BRS3-IRES-Cre mice can be propagated in the homozygous state (i.e., hemizygous males × homozygous females) without apparent effects on fertility.

### Generation of BRS3-IRES-Cre mice

A cassette encoding IRES-mnCre:GFP was inserted 3′ of the termination codon in the last coding exon of the *Brs3* gene. The 5′ arm (∼6 kb with SpeI and SalI sites at 5′ and 3′ ends, respectively) and 3′ arm (∼5.7 kb with XhoI and NotI sites at 5′ and 3′ ends, respectively) of the *Brs3* gene were subcloned from a C57BL/6 BAC clone and cloned into polylinkers of a targeting construct that contained IRES-mnCre:GFP, a frt-flanked Sv40Neo gene for positive selection, and HSV thymidine kinase and Pgk-diphtheria toxin A chain genes for negative selection. The IRES-mnCre:GFP cassette has an IRES, a Myc-tag, and nuclear localization signals at the N terminus of Cre recombinase, which is fused to green fluorescent protein followed by an SV40 polyadenylation. The construct was electroporated into G4 ES cells (C57BL/6 × 129 Sv hybrid), and correct targeting was determined by Southern blot of DNA digested with KpnI using a ^32^P-labeled probe upstream of the 5′ arm of the targeting construct. Of the 75 clones analyzed, 44 were correctly targeted. One clone that was injected into blastocysts resulted in good chimeras that transmitted the targeted allele through the germline. Progeny were bred with Gt(Rosa)26Sor-FLP recombinase mice to remove the frt-flanked SV-Neo gene. Mice were then continuously backcrossed to C57BL/6 mice. BRS3-IRES-Cre;Ai14 mice showed no evidence of germline recombinase activity in 12 progeny as assessed by lack of generalized tdTomato expression. Routine genotyping is performed with the following three primers: X717 5′ CTG CCT CAA GGC AGA GCA GC (*Brs3* forward); X718 5′ CCT CTT CTT CTC TAC TTG GTG GGC (*Brs3* reverse); and X719 5′ GCT TCG GCC AGT AAC GTT AGG (IRES reverse). The wild-type allele gives a band of ∼350 bp, while the targeted allele gives a band of ∼ 270 bp after 34 cycles with 20 s annealing at 60°C.

### Stereotactic virus injection and imaging

Mice at 3–5 months of age were anesthetized with ketamine/xylazine (80/10 mg/kg, i.p.), placed in a stereotaxic instrument (Digital Just for Mouse Stereotaxic Instrument, Stoelting), and ophthalmic ointment (Puralube, Dechra) was applied. Injections were made with pulled-glass pipettes (tip diameter, 20–40 μm; inner diameter, 0.275 mm; outer diameter, 1 mm; Wilmad Lab Glass) at a visually controlled rate of 50 nl/min using an air pressure system regulator (model S48 stimulator, Grass Technologies). Virus pAAV1-CAG-FLEX-EGFP (a gift from Hongkui Zeng Allen Institute for Brain Science, Seattle WA; viral prep #51502-AAV1, Addgene; RRID:Addgene_51502; Oh et al., 2014) was injected unilaterally into the striatum (200 nl; AP, 0.74 mm; ML, 2.00; DV, −3.5), dentate gyrus (DG; 50 nl; AP, −2.00 mm; ML, 1.5 mm; DV, −2.0 mm), and DMH (50 nl; AP, −1.85; ML, 0.3; DV, −5.05 mm), with the pipette being kept in place for 5 min after each injection. Postsurgery, mice received subcutaneous sterile saline injections and analgesic (buprenorphine, 0.1 mg/kg, i.p.). BRS3-CreER mice were given tamoxifen (110 mg/kg in corn oil, i.p.) for 5 consecutive days starting a week after surgery. Approximately 1 month after surgery, mice were perfused, and brains were fixed in 10% formalin for 1 d, transferred to 30% sucrose overnight, sliced (50 μm) on a freezing microtome (Leica), collected in three series, and one series was mounted with medium containing DAPI (Prolong Gold, Thermo Fisher Scientific). Immunohistochemistry for tdTomato on postnatal day 1 (P1) and P23 BRS3-IRES-Cre;Ai14 brain sections was performed as described previously (Piñol et al., 2018) with the exception of using 5% normal horse serum and a 2 h blocking incubation before overnight incubation with primary antibody. Slides were imaged with a VS120 slide scanner and OlyVIA software (Olympus) and a Axio Observer Z1 microscope (Zeiss) with a 10× objective, Zeiss 700 confocal hardware, and Zen software (Zeiss). Brain regions were assigned according to the study by Franklin and Paxinos (2007).

### Parabrachial nucleus neuron projection tracing

Male BRS3-IRES-Cre mice were anesthetized with isoflurane and placed on a robotic stereotaxic frame (Neurostar). AAV-1EF1a-DIO-YFP and AAV1-EF1a-DIO-synaptophysin:mCherry were injected bilaterally into the parabrachial nucleus (PBN; 200 nl; AP, −4.8 mm; ML, ±1.4 mm; DV, −3.5 mm) at a rate of 0.1 μl/min for 2 min with the needle left in place for the following 5 min. After surgery, mice were allowed to recover for ∼3 weeks. Following recovery, mice were anesthetized with phenytoin/pentobarbital and perfused with PBS, pH 7.4, followed by 4% paraformaldehyde (PFA) in 0.1 m phosphate buffer, pH 7.4. Brains were removed and placed in 4% PFA to postfix for 24 h and were subsequently placed in 30% sucrose for several days before being embedded in OCT compound and stored at −80°C. Coronal sections (35 μm) were cut on a cryostat (Leica Microsystems) and collected in cryoprotectant for long-term storage at −20°C.  To enhance the signal, sections were washed three times in PBS for 5 min and incubated in a blocking solution [3% normal donkey serum in PBS with Tween 20 (PBST)] for 1 h at room temperature. Sections were incubated overnight at 4°C in PBST with the primary antibodies chicken-anti-GFP (1:10,000; catalog #ab13970, Abcam) and rabbit-anti-DsRed (1:1000; catalog #632496, Takara Bio). The following day, sections were washed three times in PBS and then incubated for 1 h in PBS with the secondary antibodies Alexa Fluor 488 donkey anti-chicken and Alexa Fluor 594 donkey anti-rabbit (1:500; Jackson ImmunoResearch). Sections were then washed three times in PBS, mounted onto glass slides, and coverslipped with Fluoromount-G with DAPI (Southern Biotech). Images were acquired using a Keyence BZ-X700 microscope and an Olympus confocal microscope (model FV-1200, Olympus).

### RNA analysis

Male mice, 5–6 months old, were killed with CO_2_. Brains were removed and put on an ice-chilled brain matrix, and 1 mm coronal brain slices were cut with a razor blade and transferred to a microscope slide. The dorsal striatum (0–1 mm from bregma), and hippocampus and hypothalamus (both −1 to −2 mm from bregma) were microdissected with a scalpel and immediately frozen on dry ice and stored at −80°C until processed for RNA. The hypothalamus included DMH, ventromedial hypothalamus (VMH), Arc, lateral hypothalamus (LH), and PVH. RNA from microdissected tissue was isolated as reported (Xiao et al., 2015) and quantitated using QuantStudio 7 Flex Real-Time PCR System (Applied Biosystems), normalized to TATA-box binding protein (*Tbp*). The primers are as follows: *Brs3*, x575 (5′-GCACCCTGAACATACCGACT) and x576 (5′-ACAGGAGATGATTCGGCAAC); *Cre*, x760 (5′-ATGCTTCTGTCCGTTTGCCG) and x761 (5′-GACCGACGATGAAGCATGTT); *tdTomato*, x762 (5′-CCCGCCGACATCCCCGACTA) and x763 (5′-GGGTCACGGTCACCACGCC); *Mc4r*, x581 (5′-ATCTGTAGCTCCTTGCTCGC) and x582 (5′-TGCAAGCTGCCCAGATACAA); and *Tbp*, x764 (5′-TTTGTGCCAGATACATTCCG) and x765 (5′-AACAATTTACAAGCTGCGTTT).

### Mouse phenotyping

BRS3-IRES-Cre and littermate control mice were singly housed at 9 weeks of age and placed on a HFD at 11 weeks of age for metabolic studies, as described previously (Xiao et al., 2017). Another cohort of BRS3-IRES-Cre and littermate control mice was singly housed and maintained on chow diet and was used for measuring core body temperature (Tb) by telemetry as described previously (Xiao et al., 2017). Effects of MK-5046 on food intake and Tb were evaluated as reported previously (Xiao et al., 2020), with the exception that for testing the effect of MK-5046 on food intake mice were fasted for 5 h and dosed 30 min before lights off, and chow intake was measured for the first 2 h after the entry of dark phase.

### Single-cell RNA analysis

Single-cell or single-nucleus count matrices were downloaded from GEO and analyzed with R (version 4.0.2). The total number of cells and number of cells with at least one detectable *Brs3* transcript were evaluated for each dataset. True single-cell RNA (scRNA) *Brs3* positivity rates were estimated from both raw and normalized data by calculating the Poisson mean that would produce the observed ratio of (cells with one *Brs3* transcript)/(cells with greater than one *Brs3* transcript) and of (cells with one *Brs3* transcript)/(cells with two *Brs3* transcripts). *Brs3* expression in the dentate gyrus was visualized using dataset C [from the Linarsson Lab (http://linnarssonlab.org/dentate/); Hochgerner et al., 2018].

In the Arc-ME dataset GSE93374 (Campbell et al., 2017), of the 13,079 neurons, 552 expressed at least one *Brs3* transcript, and these neurons were clustered using Seurat (version 3.2.0; Butler et al., 2018; Stuart et al., 2019). Raw counts were normalized, scaled, and the 2000 most variable genes were used as input for principal component analysis. A resolution of 0.6 and 20 principal components were used for the clustering analysis, which was visualized with t-SNE (t-distributed stochastic neighbor embedding). The resolution (0.4–1.0) and principal components (10–30) were varied to confirm the robustness of the clustering. Neither condition (e.g., diet, sex, fasted state) nor batch were major drivers of the clustering. Differentially expressed genes (DEGs) and cluster marker genes were identified using the Wilcoxon rank-sum test. The top five DEGs for each cluster (based on average log_2_-fold change) were visualized by heatmap. Cluster names were assigned with one or more significantly enriched marker genes (average log_2_-fold change, >1.4; false discovery rate-adjusted *p* value, <7.5E-41), with the exception of one “unassigned” cluster in which all DEGs had an average log_2_-fold change of ≤0.71.

## Results

### Reporter expression in BRS3-IRES-Cre;Ai14 mice

**Figure 1.**
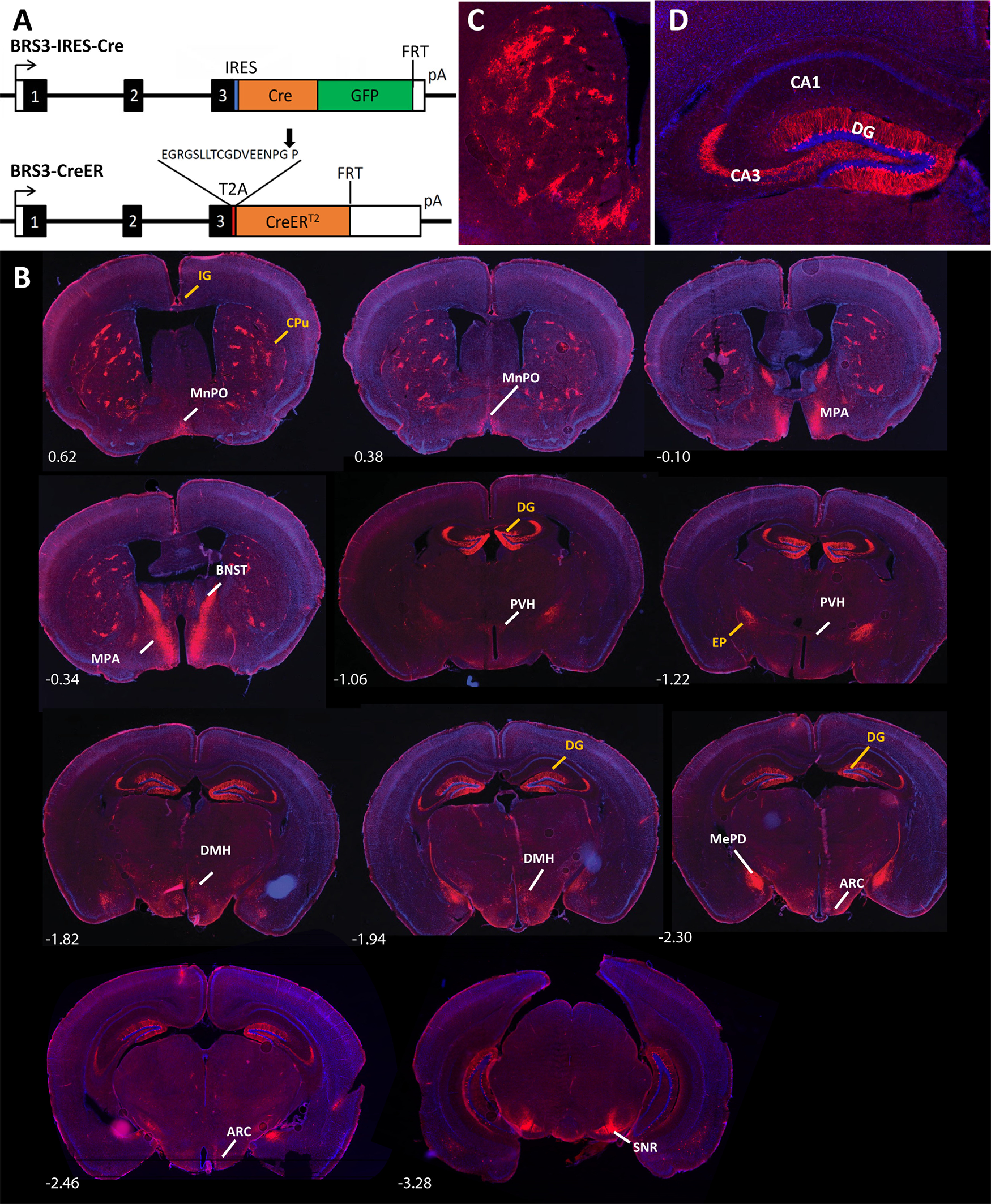
Reporter expression pattern in BRS3-IRES-Cre mice. ***A***, Schematic of the BRS3-IRES-Cre (top) and BRS3-CreER (bottom) alleles. The three exons are numbered with BRS3 coding sequences in black, untranslated regions in white, IRES in blue, T2A in red (with cleavage point indicated by an arrow), Cre in orange, and GFP in green. ***B***, Reporter expression in coronal sections from 5-month-old male BRS3-IRES-Cre;Ai14 mice at the indicated level from bregma. Labels in yellow denote reporter detected in BRS3-IRES-Cre but not BRS3-CreER mice and white labels are sites expressing reporter in both lines. Arc, Arcuate hypothalamic nucleus; DMH, dorsomedial hypothalamic nucleus. The EP and SNR fluorescence is from fibers; the rest are from cell bodies. ***C***, ***D***, Striatum (***C***) and hippocampus (***D***) at higher magnification. CA1, field CA1; CA3, field CA3.

There are currently two BRS3-Cre driver mouse lines available, one constructed by inserting a Cre recombinase sequence just after the stop codon of the *Brs3* locus (BRS3-IRES-Cre) and the other with the insertion of T2A-CreERT2 at the stop codon (BRS3-CreER; Fig. 1*A*). In BRS3-IRES-Cre mice, the Cre recombinase is constitutively active (Palmiter, 2018), whereas in BRS3-CreER mice, tamoxifen administration provides temporal control of the recombinase activity (Piñol et al., 2018). Both drivers should express *Cre* with the same pattern as wild-type BRS3, and the BRS3-CreER mice express a Cre-dependent reporter with a pattern matching that of *Brs3* mRNA (Piñol et al., 2018). The only previous information for BRS3-IRES-Cre mice is that the Cre-dependent reporter is expressed in the PBN (Palmiter, 2018). The GFP in BRS3-IRES-Cre mice was not detected, even using immunohistochemistry, presumably because of very low levels of expression of this receptor.

Here we report the tdTomato expression pattern in 5-month-old BRS3-IRES-Cre;Ai14 mice in which tdTomato expression from the Ai14 gene is Cre dependent. These mice express the reporter in multiple hypothalamic regions [median POA (MnPO); medial POA (MPA); arcuate nucleus (Arc); LH; dorsal hypothalamic area] and other brain regions [striohypothalamic nucleus; bed nucleus of the stria terminalis (BNST); posterodorsal medial amygdala (MePD); PBN; and fibers in the substantia nigra reticular (SNR); Fig. 1*B–D*, Extended Data Fig. 1-1]. All of these regions overlap with the expression pattern in BRS3-CreER;Ai14 mice. The BRS3-IRES-Cre also labeled additional cell bodies [indusium griseum (IG); caudate putamen or striatum (CPu); DG] and fibers [endopeduncular nucleus (EP)]. In summary, BRS3-IRES-Cre mice have reporter expression in all regions seen in BRS3-CreER mice, plus some additional sites.

10.1523/ENEURO.0252-21.2021.f1-1 Figure 1-1 A listing of the expression sites. Download Figure 1-1, DOCX file.

### Lineage tracing in BRS3-IRES-Cre mice

**Figure 2.**
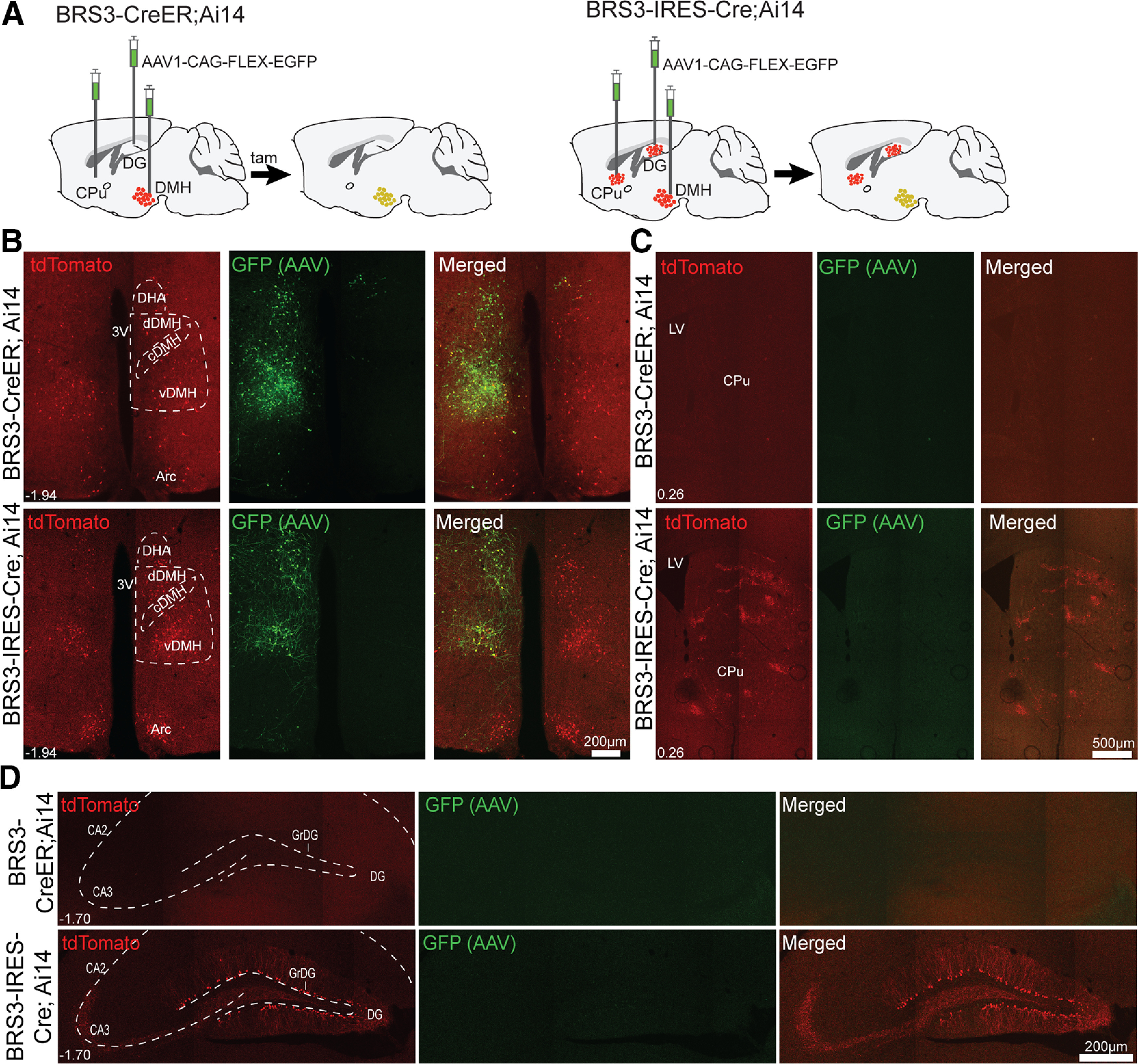
Current compared with lineage *Brs3* reporter expression. ***A***, BRS3-CreER;Ai14 and BRS3-IRES-Cre;Ai14 (3- to 5-month-old) mice were injected unilaterally with AAV1-CAG-FLEX-EGFP carrying Cre-dependent GFP. The presence of tdTomato indicates either prior or current Cre activity, while GFP indicates Cre activity at the time of virus injection/tamoxifen treatment, 2 weeks before being killed. ***B–D***, DMH (***B***), striatum (***C***), and hippocampus (***D***). Bregma levels are indicated.

The additional reporter expression in BRS3-IRES-Cre mice could be from the following: (1) Cre expression in regions not normally expressing *Brs3* (ectopic sites); (2) higher Cre expression in sites that do express *Brs3* (eutopic sites); and/or (3) lineage effects with Cre expression in a precursor cell (or prior expression in the current cell). To distinguish among these possibilities, we injected a virus carrying a Cre-dependent GFP construct into three brain regions of BRS3-IRES-Cre;Ai14 and BRS3-CreER Ai14 mice. Cre activity is required at the time of virus injection/tamoxifen treatment for GFP expression while either prior or current Cre activity will produce a tdTomato signal (Fig. 2*A*).

In 3- to 5-month-old BRS3-CreER;Ai14 mice, virus-derived GFP was present in the DMH, but not the hippocampus or striatum (Fig. 2*B–D*). Virus-derived reporters have a higher copy number and are typically expressed at higher levels than host genome-derived reporters. Thus, lower eutopic Cre activity in BRS3-CreER;Ai14 mice likely does not explain the lack of GFP in the hippocampus and striatum.

BRS3-IRES-Cre;Ai14 mice showed the same viral GFP expression pattern (present in DMH, but not in hippocampus or striatum) as the BRS3-CreER;Ai14 mice. Thus, at the time of viral injection, there was no active Cre recombinase in the hippocampus or striatum. As expected, the BRS3-IRES-Cre;Ai14 mice expressed tdTomato in all three regions. These results suggest that *Brs3* is expressed earlier in hippocampal and striatal neuron development, but no longer at 3–5 months of age, consistent with lineage tracing in BRS3-IRES-Cre mice.

**Figure 3.**
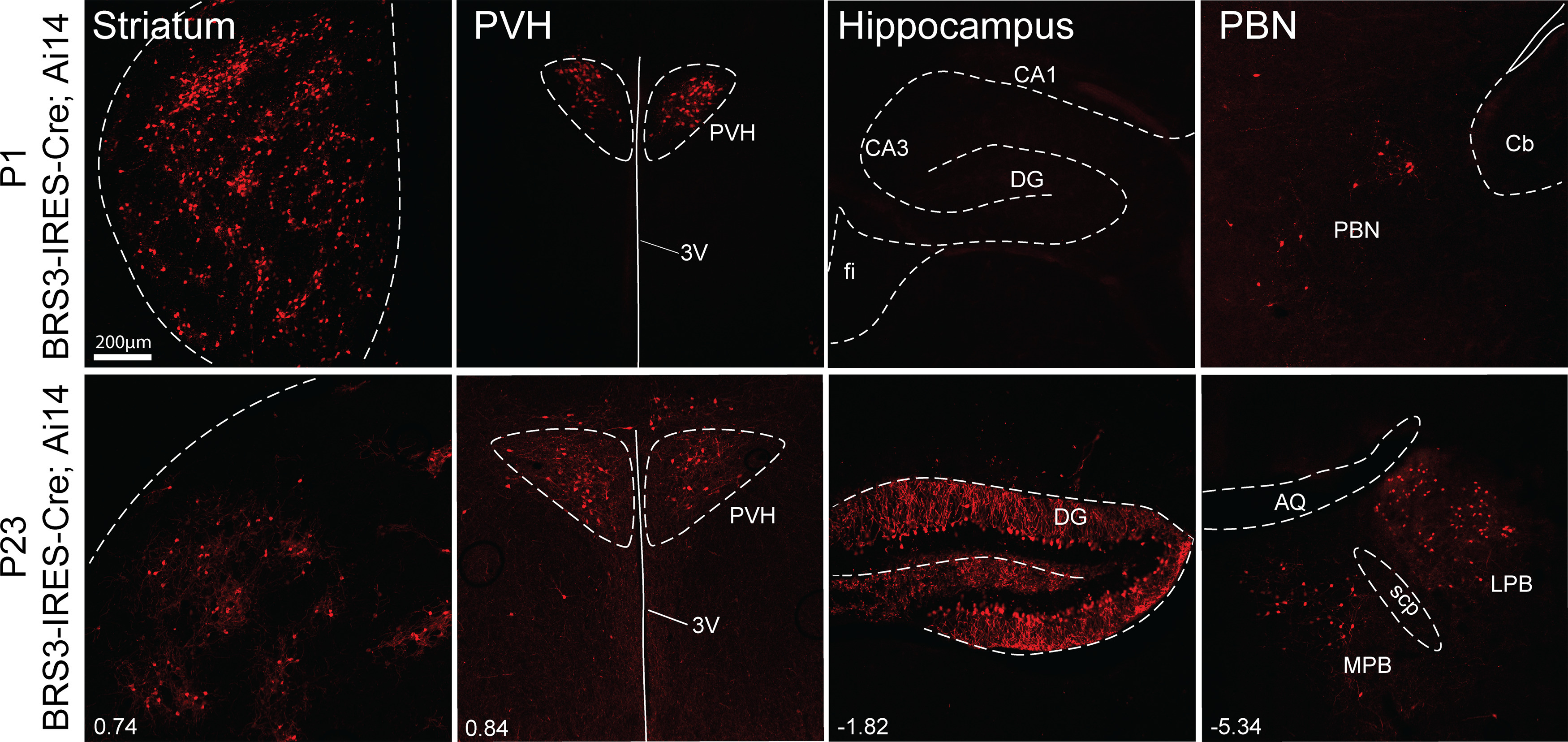
BRS3-IRES-Cre-driven reporter expression in young mice. tdTomato reporter expression in BRS3-IRES-Cre;Ai14 mice at P1 (top row) and P23 (bottom row). Scale bar, 200 μm. Bregma levels are indicated. 3V, Third ventricle; fi, fimbria; Cb, cerebellum; AQ, cerebral aqueduct; scp, superior cerebellar peduncles; LPB, lateral parabrachial nucleus; MPB, medial parabrachial nucleus.

Examination of genomic reporter expression in BRS3-IRES-Cre;Ai14 mice on P1 demonstrated expression in the striatum, PVH, and PBN, but not the hippocampus (Fig. 3). By P23, all four of these regions contained tdTomato reporter. Together, the results suggest that striatal *Brs3* expression is on at (or before) P1 and turns off sometime before adulthood. Hippocampal *Brs3* expression is initially off (P1) and is turned on at (or before) P23.

### *Brs3* expression as a defining feature of neuronal populations

**Figure 4.**
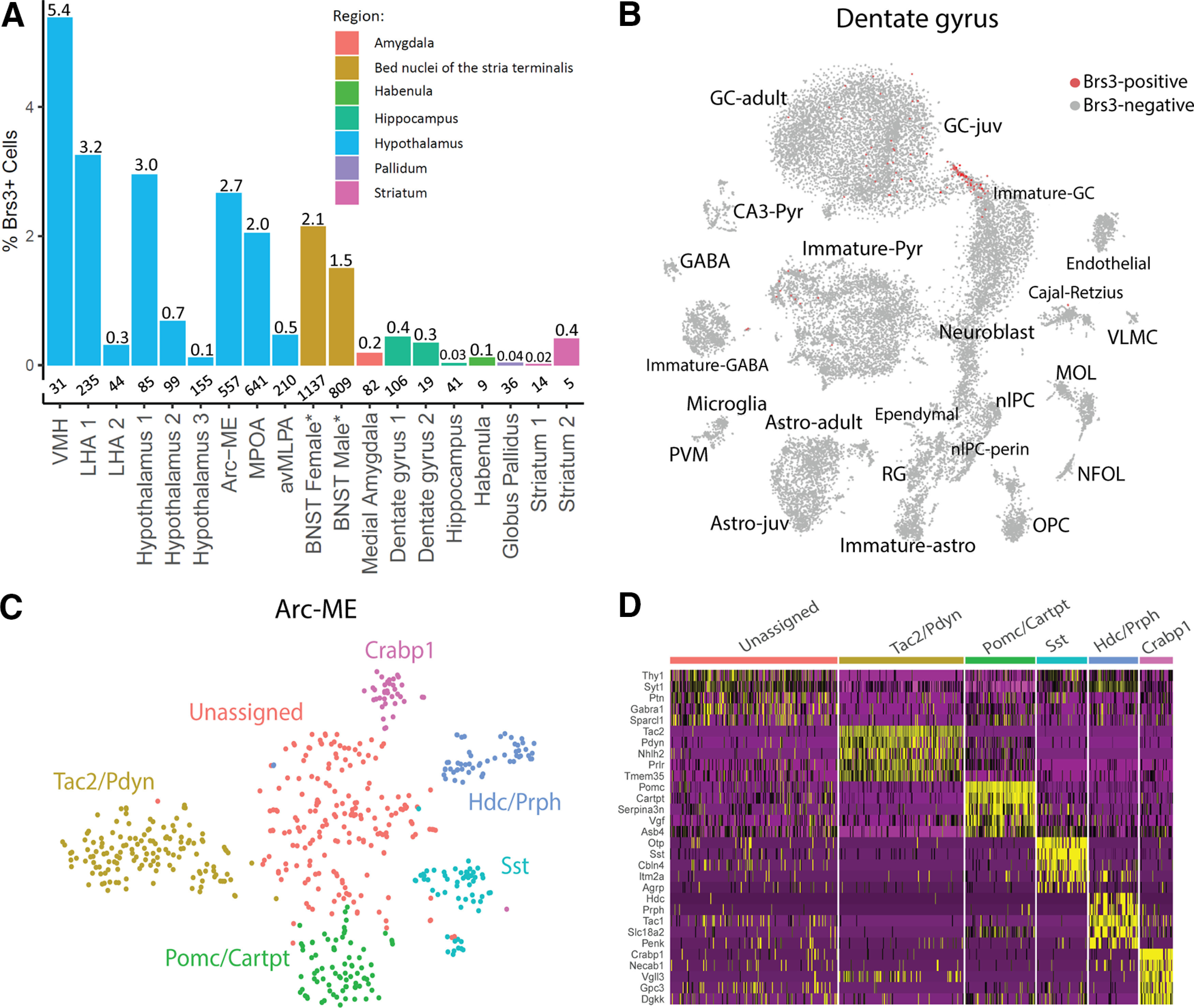
*Brs3* expression in scRNA sequence datasets. ***A***, Percentage of neurons expressing *Brs3* in datasets with ≥5 *Brs3*-positive cells (Extended Data Fig. 4-1, lists of datasets with fewer *Brs3*-positive cells). The number of detected *Brs3*-expressing cells is indicated below each bar. An *Total cells, not just neurons. Data are from the following, in order: GSE143818, GSE125065, GSE130597, GSE74672, GSE132355 GSE87544, GSE93374, GSE113576, GSE149344 GSE126836, GSE126836, GSE124061, GSE104323, GSE95315, GSE116470, GSE146983, GSE116470, GSE116470, and GSE82187. ***B***, Dentate gyrus *Brs3* expression from http://linnarssonlab.org/dentate/ (Hochgerner et al., 2018). ***C***, t-SNE plot of the *Brs3*-expressing Arc-ME neurons. ***D***, Heatmap of expression of the top five enriched genes in each cluster. The *Brs3* clusters correspond to the original clusters as follows: *Hdc/Prph*, n01 and n02; *Tac2/Pdyn*, n20; *Pomc/Cartpt*, n15 > n14; *Crabp1*, n26; and *Sst*, n23 > n12, and n13.

We next compared the *Brs3* expression patterns determined from the Cre reporters with that in published mouse single-cell RNA datasets (Fig. 4*A*, Extended Data Fig. 4-1) (Guo Q, Li JYH, 2019; Huang KW, Sabatini BL, 2020; Huang et al, 2019; Huisman et al., 2019; Mizrak et al., 2020; Tepe et al., 2018; Van Hove et al., 2019; Wizeman et al., 2019). Generally, the *Brs3* scRNA expression/nonexpression pattern matched that in the BRS3-Cre mice. For example, some striatal neurons were *Brs3*-positive [5 of 368 or 1.4% (Gokce et al., 2016) and 14 of 72,897 or 0.019% (Saunders et al., 2018)]. In a hippocampus developmental series, *Brs3* was expressed in immature and juvenile granule cell neurons of the dentate gyrus (Hochgerner et al., 2018; Fig. 4*B*).

10.1523/ENEURO.0252-21.2021.f4-1 Figure 4-1 Datasets with fewer *Brs3*-positive cells are listed. See studies by Saunders et al. (2018), Tepe et al. (2018), Guo and Li (2019), Huang et al., 2019), Huisman et al. (2019), Van Hove et al. (2019), Wizeman et al. (2019), Huang and Sabatini (2020), and Mizrak et al. (2020). Download Figure 4-1, XLS file.

No scRNA dataset had >5% BRS3-positive cells. In scRNA datasets with >100 BRS3-positive cells (GSE93374, GSE104323, GSE113576, GSE125065, and GSE149344), the positivity was because of a single detected *Brs3* transcript/cell in 69%, 86%, 68%, 63%, and 81% of the cells, respectively, suggesting that *Brs3* false-negative findings are likely. Assuming a Poisson distribution, the actual *Brs3*-positive percentages are 1.1-fold to 3-fold of the nominal percentages, depending on the dataset, normalization, and assumptions used in calculating event rates.

To explore the impact of the *Brs3* false negative findings, we studied a dataset of Arc-ME scRNA sequences (Campbell et al., 2017), chosen for its large number of *Brs3*-positive neurons (4.2% of total). Clustering only the 552 *Brs3*-positive neurons yielded six clusters, five of which mapped to nine of the clusters reported in the study by Campbell et al. (2017; Fig. 4*C*,*D*). Only two of the original clusters had high enough detected *Brs3* positivity (46% in n01 and 42% in n02) to indicate that *Brs3* might be present in all cluster members. Thus, removing the *Brs3* detection bias did not improve the clustering analysis.

### Projections of PBN^BRS3^ neurons

**Figure 5.**
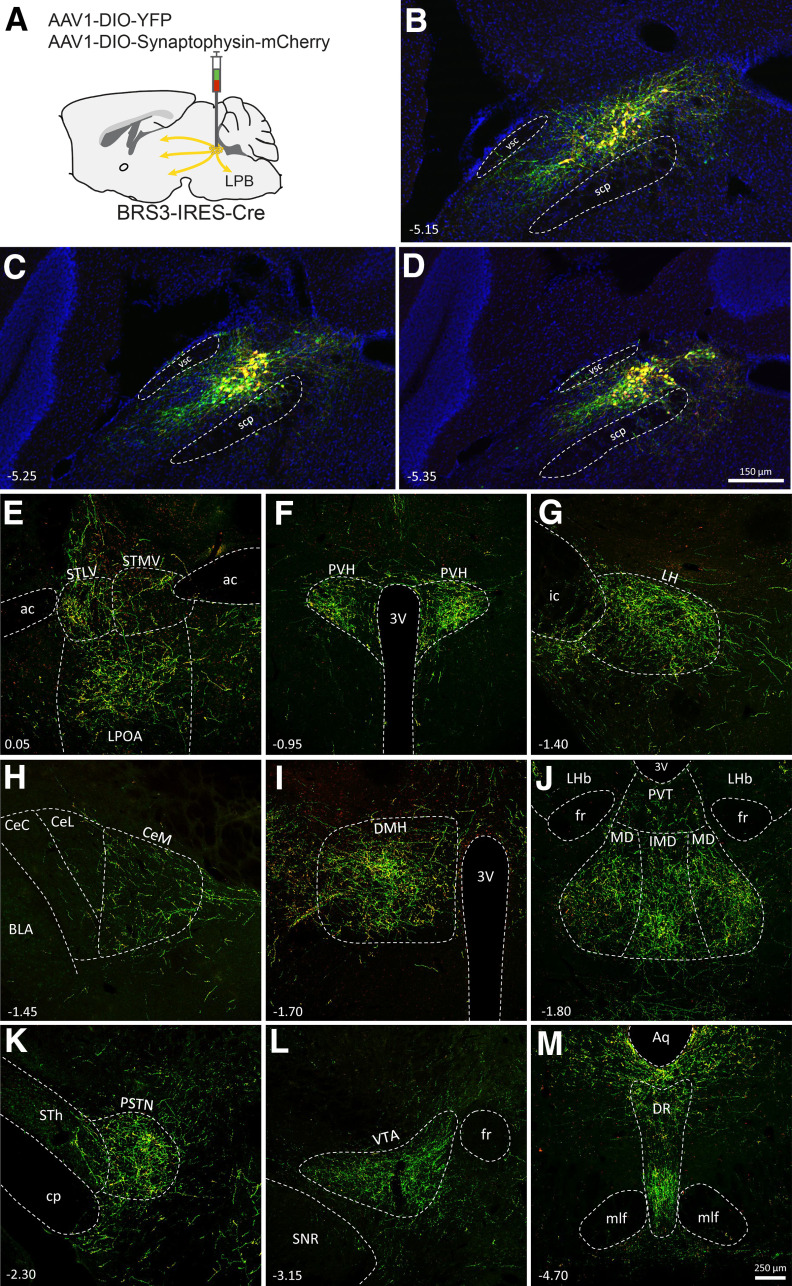
Brs3-expressing neurons in the lateral parabrachial nucleus project axons to multiple forebrain regions. AAV1-DIO-YFP and AAV1-DIO-synaptophysin:mCherry were coinjected into the PBN of BRS3-IRES-Cre mice. Several weeks later mice were perfused, sectioned, and then sections throughout the brain were treated with antibodies against YFP and DsRed to detect expression. Yellow reveals regions where both fluorescent proteins are present in cell bodies and terminals, whereas only green (e.g., VTA) reveals fibers of passage. ***A***, Diagram showing injection site. ***B–D***, viral expression at three bregma levels of the PBN. ***E–M***, Fluorescent fibers in preoptic area (***E***); paraventricular nucleus (***F***); lateral hypothalamus (***G***); central nucleus of the amygdala (***H***); dorsal medial hypothalamus (***I***); intermediodorsal thalamus (***J***); parasubthalamic nucleus (***K***); ventral tegmental nucleus (***L***); and dorsal raphe (***M***). Bregma levels are indicated by numbers.

The PBN receives diverse sensory inputs and integrates and transmits this information to many forebrain regions (Palmiter, 2018; Chiang et al., 2019). As a step in characterizing PBN^BRS3^ neurons, the BRS3-IRES-Cre mouse was used to trace the projections from PBN^BRS3^ neurons (Fig. 5). Projections were observed to the preoptic area (lateral POA), hypothalamus (PVH, LH, DMH, principal sensory trigeminal nucleus), amygdala (BNST, central nucleus of the amygdala), thalamus [mediodorsal thalamus (MD), ipsilateral MD], and dorsal raphe; the VTA signal is likely caused by fibers of passage because synaptophysin expression in the region is very low. Most of these nuclei have a role in regulating energy homeostasis.

### Metabolic phenotype of BRS3-IRES-Cre mice

**Figure 6.**
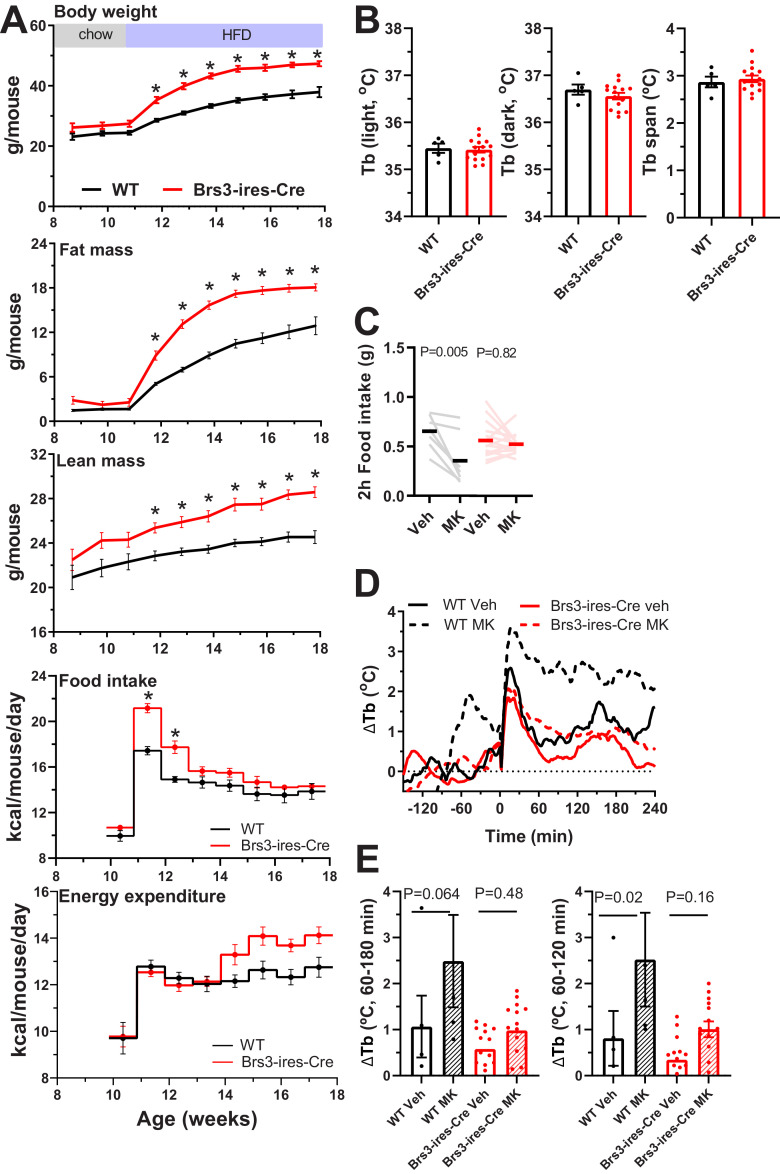
Metabolic phenotype of BRS3-IRES-Cre mice. ***A***, Effect of a high-fat diet in male BRS3-IRES-Cre and littermate control mice (*n* = 8/group) on body weight, fat mass, lean mass, food intake, and energy expenditure determined by mass balance (Ravussin et al., 2013). Data are the mean ± SEM; *adjusted *p* < 0.05 from two-way ANOVA (Extended Data Fig. 6-1, with Šídák's multiple-comparisons test). ***B–D***, Baseline core body temperature (***B***), effect of MK-5046 (10 mg/kg, i.p., given at onset of the dark cycle in 5 h fasted mice) on food intake (***C***), and effect of MK-5046 (10 mg/kg, i.p., at 10:00 A.M. in overnight-fasted mice) on body temperature (***D***). ***E***, Tb changes from baseline (−150 to −30 min) to 60–180 min and 60–120 min after dosing in a crossover design. The *p* values are from two-way ANOVA with Šídák's multiple-comparisons test, see Extended Data Figure 6-1. Mice in ***B–E*** are chow-fed males (*n* = 5–15/group). In ***D***, data are the mean with the SEM omitted for visual clarity.

The BRS3-CreER allele is hypomorphic (Piñol et al., 2018), so the effect of the BRS3-IRES-Cre allele on metabolic physiology was examined. Male BRS3-IRES-Cre mice fed a chow diet were nonsignificantly heavier than control littermates by 1.8 g at 12 weeks (26.6 ± 0.6 vs 24.8 ± 0.7 g, *p* = 0.087; *n* = 9–15/group) and by 1.5 g at 14 weeks (27.5 ± 0.5 vs 26.1 ± 4.4 g, *p* = 0.10; *n* = 7–15/group). In an independent cohort, male BRS3-IRES-Cre mice switched to a high-fat diet gained more weight and adiposity than littermate controls, initially with increased food intake and unchanged energy expenditure (Fig. 6*A*, Extended Data Fig. 6-1). The phenotype is similar to the BRS3-null mice (Ohki-Hamazaki et al., 1997b) and BRS3-CreER mice (Piñol et al., 2018).

10.1523/ENEURO.0252-21.2021.f6-1 Figure 6-1 The *p* values are from two-way ANOVA with Šídák's multiple-comparisons test. Download Figure 6-1, XLS file.

A robust thermal phenotype of BRS3-null mice is an increased Tb span (defined as the difference between the 95th and 5th Tb percentiles over integral multiples of 24 h intervals; Xiao et al., 2017). The Tb span was not increased in the BRS3-IRES-Cre mice compared with wild-type littermates, and, as expected, there was no difference in either light- or dark-phase Tb (Fig. 6*B*). However, the BRS3-IRES-Cre mice lost the suppression of food intake elicited by treatment with a BRS3 agonist, MK-5046 (Fig. 6*C*), and the BRS3 agonist effect of increasing light-phase Tb (Guan et al., 2011) was blunted in the BRS3-IRES-Cre mice (Fig. 6*D*,*E*). These results indicate that the BRS3-IRES-Cre allele is a hypomorph, with a phenotype milder than the complete BRS3-null mice.

### Reduced *Brs3* mRNA levels in BRS3-IRES-Cre mice

**Figure 7.**
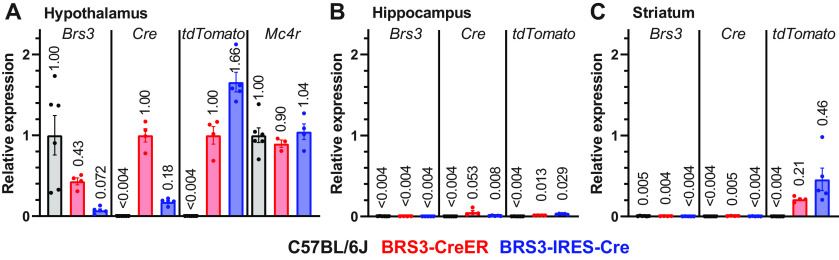
mRNA levels in hypothalamus, hippocampus, and striatum of the two BRS3-Cre mouse models. mRNA expression was quantified by RT-PCR in hypothalamus (***A***), hippocampus (***B***), and striatum (***C***) of control (C57BL/6J, black), BRS3-CreER (red), and BRS3-IRES-Cre (blue) mice at 5–6 months of age. In all samples, *Brs3* and *Mc4r* mRNA levels are normalized to the C57BL/6J hypothalamus, and *Cre* and *tdTomato* mRNA levels are normalized to the BRS3-CreER hypothalamus. *Mc4r* mRNA levels are a dissection and technical control. Mean values are indicated above each bar. Expression below the limit of detection is indicated as <0.004 (*n* = 4–6/group).

To better understand the phenotype of the BRS3-IRES-Cre mice, we measured RNA levels in 5- to 6-month-old, wild-type (C57BL/6J), BRS3-CreER;Ai14, and BRS3-IRES-Cre;Ai14 (Fig. 7) mice. Hypothalamic *Brs3* mRNA levels in BRS3-CreER mice were 43 ± 4% of wild-type mice and 7.2 ± 1.7% in BRS3-IRES-Cre mice. The relative RNA levels likely explain the more severe metabolic phenotype in the BRS3-IRES-Cre mice.

In both BRS3-Cre drivers, BRS3 and Cre are encoded in a single mRNA, and, as expected, the *Brs3*/*Cre* mRNA ratios were similar in the BRS3-CreER;Ai14 and BRS3-IRES-Cre;Ai14 mice (2.3 and 3.4, respectively). *Cre* mRNA in the hypothalamus of BRS3-IRES-Cre mice is 18% of the level in BRS3-CreER mice. Interestingly, *tdTomato* mRNA levels in the hypothalamus of BRS3-IRES-Cre mice were 166% of that in BRS3-CreER mice, suggesting that there are more tdTomato-expressing neurons (and not more *tdTomato* mRNA/cell since both mice use the same reporter locus) in BRS3-IRES-Cre;Ai14 mice. Possible mechanistic explanations for increased tdTomato-positive neurons are lineage effects in BRS3-IRES-Cre;Ai14 mice and/or incomplete activation of Cre by tamoxifen in BRS3-CreER;Ai14 mice.

In the hippocampus, very low levels of *Cre* and *tdTomato* mRNA were detected in both BRS3-CreER;Ai14 and BRS3-IRES-Cre;Ai14 mice. In the striatum, low levels of *tdTomato* mRNA (BRS3-IRES-Cre > BRS3-CreER) were observed. Apparent differences between mRNA levels and fluorescence signal may be because of translational efficiency and/or protein stability, as protein levels were not quantitated.

## Discussion

The design of genomic reporter/driver mice has evolved from random insertion of small-plasmid DNAs to random insertion of larger DNAs (e.g., bacterial artificial chromosomes), and to targeted insertion into the endogenous locus, recently with high efficiency using CRISPR/Cas9 technology. Thus, as with the BRS3-Cre mice, one can now reliably produce driver/reporter alleles that have a high likelihood of correctly tracking the expression pattern of the target gene, a major advance [see (Song and Palmiter, 2018; Luo et al., 2020) for some cautions].

Lineage effects in nonconditional systems are widely recognized (Daigle et al., 2018), but infrequently characterized in detail [exceptions are proopiomelanocortin (POMC; Padilla et al., 2010) and GFAP (Ganat et al., 2006)]. The differences between the two BRS3-Cre drivers illustrate the value of having available both inducible and constitutive recombinases.

The large doses of tamoxifen required for efficient Cre recombinase activity can reduce body weight and cause loss of adipose tissue (Ye et al., 2015). Also, *Brs3* expression is sex dimorphic (Xu et al., 2012; Chen et al., 2019), and *Brs3* neurons are involved in sex-dimorphic behaviors that may be regulated by estrogen receptors (Moffitt et al., 2018). Thus, the ability to select inducible, tamoxifen-dependent, and/or constitutive tamoxifen-independent driver mice improves experimental design.

The *Brs3* locus is unforgiving for expressing a recombinase/reporter allele since *Brs3* is on the X chromosome, undergoes X-inactivation, and is expressed at a low level. Thus, modified alleles must preserve BRS3 function while optimizing reporter expression. Unfortunately, both BRS3-Cre alleles are hypomorphs, with more function in BRS3-CreER than BRS3-IRES-Cre allele (Table 1, summary), and the metabolic effects of the deficiency should be considered when the mice are used.

**Table 1:** Summary of mouse *Brs3* allele phenotypes

		C57BL/6J	BRS3-CreER	BRS3-IRES-Cre	BRS3-null
Body weight		Reference	Increased	Increased	Increased
Body temperature span		Reference	No change	No change	Increased
Reporter expression in adult Ai14 mice	Hypothalamus		Yes	Yes	
	Parabrachial nucleus		Yes	Yes	
	Hippocampus		No	Yes	
	Striatum		No	Yes	
Hypothalamus *Brs3* RNA		100%	43%	7%	None
Food intake inhibition by BRS3 agonist, MK-5046		Yes	Intact	Lost	Lost
Body temperature increase by BRS3 agonist, MK-5046		Yes	Intact	Blunted	Lost

BRS3-IRES-Cre observations are presented in Results; BRS3-CreER data are from Results and the study by Piñol et al. (2018); and the BRS3*-*null phenotype is from the studies by Ohki-Hamazaki et al. (1997b), Guan et al. (2011), and Xiao et al. (2017), among others.

Each allele produces a single mRNA that encodes both BRS3 and Cre. The T2A sequence in BRS3-CreER causes a ribosome skip, with the downstream protein being produced in similar amounts to the upstream one, although the upstream protein has 17 aa appended, which may affect function (Szymczak-Workman et al., 2012). In the BRS3-IRES-Cre allele, the IRES system results in translation of the downstream Cre at a fraction of the level of the upstream gene (Mizuguchi et al., 2000). While the T2A and IRES sequences differentially affect protein levels, they do not explain the reduced *Brs3* mRNA levels, which are presumably because of mRNA instability or reduced transcription caused by the inserted genomic sequences, such as by interfering with enhancer function. It is notable that the BRS3-IRES-Cre allele produces efficient recombination despite a 93% reduction in *Brs3* mRNA levels and further reduced Cre protein because of the IRES. The effectiveness of the recombinase means that quantitative differences in *Brs3* mRNA levels, such as the sex dimorphism in the BNST and medial amygdala (Xu et al., 2012; Chen et al., 2019), are not detected in reporter mice.

The availability of characterized BRS3-Cre driver alleles facilitates investigation of BRS3 function in pancreatic islets (Feng et al., 2011), neuroendocrine tumors (Sherman et al., 2014), and certain cancers (Moreno et al., 2018; Ramos-Alvarez et al., 2019). The limited number of discrete brain nuclei expressing *Brs3* means that the BRS-Cre drivers are particularly valuable for intersectional genetic studies (Madisen et al., 2015) of the neural circuits and networks that control metabolism and other processes.

## Synthesis

Reviewing Editor: Kevin Wickman, University of Minnesota Decisions are customarily a result of the Reviewing Editor and the peer reviewers coming together and discussing their recommendations until a consensus is reached. When revisions are invited, a fact-based synthesis statement explaining their decision and outlining what is needed to prepare a revision will be listed below. The following reviewer(s) agreed to reveal their identity: Rodrigo Escartin Pérez, Laurent Gautron.

The authors present an in-depth characterization of a novel constitutive BRS3-Cre mouse line, and provide some comparison to a previously described induced BRS3-Cre driver line. Using viral tracing, metabolic phenotyping, qPCR, and neuroanatomical mapping, the authors provide evidence of reduced BRS3 expression in both lines and characterize the resulting hypofunction phenotype in the new line, which includes increased body weight. In total, the data support the contention that BRS3 contributes to the physiology of energy balance and eating behavior in mice, while reinforcing the important caveat that gene knock-in approaches often alter the expression of the endogenous gene.

The article is well-written and straightforward and includes detailed description of methods employed, and data are presented carefully and interpreted with appropriate caution. As such, this study should be a useful resource to others interested in the BRS3 system.

The only major concern raised by the reviewers pertained to statistical analysis; these concerns should be addressed in revision:

1. Data from experiments in which authors evaluated the effects of a hypercaloric diet on different variables (body weight, fat and lean tissue, and energy expenditure; Fig 6A) should be analyzed using repeated-measures 2-way ANOVA (two levels of group factor at ten repeated measures for each dependent variable). The use of multiple t-tests without a correction measure is inappropriate.

2. Data from experiments in which authors evaluated the effects of MK-5046 on food intake and change on basal body temperature (Fig 6C and 6E) should be analyzed using regular 2-way ANOVA. There are two factors: group (WT, BRS3-IRES-Cre) and treatment (Vh, MK-5049). If the results indicate a violation of ANOVA assumptions, authors should use corrected versions of calculations and the appropriate post hoc test.

A few minor concerns were also raised by the reviewers, and these should be addressed in the revision and/or critique response:

1. In Fig 1, there are no images of the brainstem included. In fact, the brainstem is not discussed at all. Was there any tomato expression in the brainstem?

2. In Fig 6, a core temperature of 35.5 Celsius during the day seems low. Do the authors have an explanation for this relatively low core body temperature?

3. Do the authors know if the levels of endogenous ligands for BRS3 (neuromedin, GRP) are altered in mice with reduced expression of BRS3?

## Author Response

page 1

1

Dear Dr Wickman,

RE: eN-TNWR-0252-21

We are pleased that our manuscript “Cre recombinase driver mice reveal lineage-dependent and -independent expression of Brs3 in the mouse brain” was judged

potentially suitable for publication in eNeuro. We have revised the manuscript to

address all of your comments and suggestions. Our responses to the reviewers’

comments are detailed below. Changes to the manuscript are shown in yellow in a

supplemental file.

Stay well and regards,

Marc

Reviewers’ Comments:

Synthesis Statement for Author (Required):

The authors present an in-depth characterization of a novel constitutive BRS3-Cre

mouse line, and provide some comparison to a previously described induced BRS3-Cre

driver line. Using viral tracing, metabolic phenotyping, qPCR, and neuroanatomical

mapping, the authors provide evidence of reduced BRS3 expression in both lines and

characterize the resulting hypofunction phenotype in the new line, which includes

increased body weight. In total, the data support the contention that BRS3 contributes

to the physiology of energy balance and eating behavior in mice, while reinforcing the

important caveat that gene knock-in approaches often alter the expression of the

endogenous gene.

The article is well-written and straightforward and includes detailed description of

methods employed, and data are presented carefully and interpreted with appropriate

caution. As such, this study should be a useful resource to others interested in the BRS3

system.

The only major concern raised by the reviewers pertained to statistical analysis; these

concerns should be addressed in revision:

1. Data from experiments in which authors evaluated the effects of a hypercaloric diet

on different variables (body weight, fat and lean tissue, and energy expenditure; Fig 6A)

should be analyzed using repeated-measures 2-way ANOVA (two levels of group factor

at ten repeated measures for each dependent variable). The use of multiple t-tests

without a correction measure is inappropriate.

Response: Thank you. We now analyze Fig 6A with 2-way ANOVA. At each timepoint,

the P value for the WT vs BRS3-IRES-Cre comparison is calculated using Šídák’s multiple

comparisons test. The figure has been updated and the ANOVA details are in new Figure

6-1. page 2

2

2. Data from experiments in which authors evaluated the effects of MK-5046 on food

intake and change on basal body temperature (Fig 6C and 6E) should be analyzed using

regular 2-way ANOVA. There are two factors: group (WT, BRS3-IRES-Cre) and treatment

(Vh, MK-5049). If the results indicate a violation of ANOVA assumptions, authors should

use corrected versions of calculations and the appropriate post hoc test.

Response: As suggested, we have analyzed Figs 6d and E with 2-way ANOVA with post

hoc comparisons using Šídák’s multiple comparisons test. The figure has been updated

and the ANOVA details are in Figure 6-1

A few minor concerns were also raised by the reviewers, and these should be addressed

in the revision and/or critique response:

1. In Fig 1, there are no images of the brainstem included. In fact, the brainstem is not

discussed at all. Was there any tomato expression in the brainstem?

Response: The second page of the supplementary table, Figure 1-1 summarizes the

brainstem expression pattern.

2. In Fig 6, a core temperature of 35.5 Celsius during the day seems low. Do the authors

have an explanation for this relatively low core body temperature?

Response: It is slightly lower than we usually observe, but we do sometimes have wild

type cohorts with similar body temperatures. An important point is that both groups

were studied at the same time, so any environmental events affected both groups.

3. Do the authors know if the levels of endogenous ligands for BRS3 (neuromedin, GRP)

are altered in mice with reduced expression of BRS3?

Response: Despite multiple de-orphanization attempts, BRS3 remains an orphan

without an identified endogenous ligand (Civelli et al., 2013). We added the following

sentence to the introduction: “BRS3 is considered an orphan receptor as its endogenous

ligand is not known; specifically it does not bind the natural ligands (gastrin-releasing

peptide and neuromedin B) for the most closely related receptors (Mantey et al., 1997),

nor does it bind bombesin, which is the frog ortholog of neuromedin B (Hirooka et al.,

2021).”

Civelli O, Reinscheid RK, Zhang Y, Wang Z, Fredriksson R, Schioth HB (2013) G protein coupled receptor deorphanizations. Annu Rev Pharmacol Toxicol 53:127-146.page 3

3

Hirooka A, Hamada M, Fujiyama D, Takanami K, Kobayashi Y, Oti T, Katayama Y,

Sakamoto T, Sakamoto H (2021) The gastrin-releasing peptide/bombesin system

revisited by a reverse-evolutionary study considering Xenopus. Sci Rep 11:13315.

Mantey SA, Weber HC, Sainz E, Akeson M, Ryan RR, Pradhan TK, Searles RP, Spindel ER,

Battey JF, Coy DH, Jensen RT (1997) Discovery of a high affinity radioligand for

the human orphan receptor, bombesin receptor subtype 3, which demonstrates

that it has a unique pharmacology compared with other mammalian bombesin

receptors. J Biol Chem 272:26062-26071
