# Supplementary material for: Cre Recombinase Driver Mice Reveal Lineage-Dependent and -Independent Expression of Brs3 in the Mouse Brain
Source: eNeuro. 2021 Aug 16;8(4):ENEURO.0252-21.2021. doi: 10.1523/ENEURO.0252-21.2021 (PMC8371926; doi:10.1523/ENEURO.0252-21.2021)
Supplement: Figure 1-1 — A listing of the expression sites. Download Figure 1-1, DOCX file. [file enu-eN-NWR-0252-21-s01.docx]

**Figure 1-1. Brain expression of *Brs3***

| **Region** | | **BRS3-IRES-Cre (tdTomato)^1^** | | **BRS3-CreER (tdTomato)^2^** | | **Mouse mRNA^3^** | | **Rat mRNA^3^** | |
| --- | --- | --- | --- | --- | --- | --- | --- | --- | --- |
| *Telencephalon* | |  | |  | |  | |  | |
| **Olfactory bulb** | | _−_^4^ | | − | | − | | − | |
| **Cerebral cortex** | |  | |  | |  | |  | |
| Intermediate entorhinal cortex | | + | | + | | − | | − | |
| **Hippocampal formation** | |  | |  | |  | |  | |
| Dentate gyrus | | ++++ | | − | | − | | − | |
| Indusium griseum | | + | | − | | NR | | NR | |
| **Amygdala and Bed nucleus of the stria terminalis** | |  | |  | |  | |  | |
| Anterior amygdaloid area (AA) | | − | | − | | − | | + | |
| Interstitial n. of the posterior limb of the anterior commissure (IPAC) | | + | | − | | + | | ++ | |
| Basomedial amygdaloid n., anterior part (BMA) | | + | | + | | − | | + | |
| Basolateral amygdaloid n., anterior part (BLA) | | − | | − | | − | | −/+ | |
| Amygdalostriatal transition area (Astr) | | − | | − | | − | | + | |
| Anterior cortical amygdaloid nucleus (ACo) | | + | | − | | − | | + | |
| Posteromedial cortical amygdaloid n. (PMCo) | | − | | − | | − | | −/+ | |
| Central amygdaloid n., lateral division (CeL) | | −/+ | | − | | −/+ | | ++ | |
| Central amygdaloid n., central division(CeC) | | − | | − | | + | | ++ | |
| Central amygdaloid n., medial division (CeM) | | −/+ | | + | | + | | ++ | |
| Medial amygdaloid n., anterodorsal part (MeAD) | | −/+ | | − | | ++ | | ++ | |
| Medial amygdaloid n., anteroventral part (MeAV) | | −/+ | | − | | + | | + | |
| Medial amygdaloid n., posterodorsal part (MePD) | | ++++ | | ++++ | | + | | ++ | |
| Medial amygdaloid n., posteroventral part (MePV) | | −/+ | | − | | + | | +/++ | |
| Bed n. of the stria terminalis, medial division, anterior part (STMA) | | + | | + | | + | | +/++ | |
| Bed n. of the stria terminalis, medial division, posteromedial part (STMPM) | | ++++ | | +++ | | + | | + | |
| Bed n. of the stria terminalis, medial division, anteromedial part (STMAM) | | ++ | | + | | ++ | | − | |
| Bed n. of the stria terminalis, medial division, ventral part (STMV) | | ++ | | + | | + | | + | |
| Bed n. of the stria terminalis, medial division, posterolateral part (STMPL) | | + | | + | | + | | + | |
| Bed n. of the stria terminalis, medial div., posterointermediate part (STMPI) | | ++ | | ++ | | ++ | | −/+ | |
| Bed n. of the stria terminalis, lateral division, posterior part (STLP) | | −/+ | | + | | − | | + | |
| Bed n. of the stria terminalis, lateral division, intermediate part (STLI) | | −/+ | | − | | − | | −/+ | |
| N. of the stria medullaris (SM) | | ++/+++ | | − | | + | | − | |
| **Striatum** | |  | |  | |  | |  | |
| Accumbens n., core (AcbC) | | ++ | | + | | ++ | | ++ | |
| Accumbens n., shell (AcbSh) | | + | | + | | + | | + | |
| Lateral accumbens shell (LAcbSh) | | − | | − | | + | | + | |
| Dorsal endopiriform n. (DEn) | | − | | − | | − | | −/+ | |
| Caudate putamen (CPu) | | +++ | | − | |  | | − | |
| *Diencephalon* | |  | |  | |  | |  | |
| **Thalamus** | |  | |  | |  | |  | |
| Zona incerta (ZI) | | − | | − | | + | | + | |
| Medial habenular n. (MHb) | | ++ | | − | | − | | ++++ | |
| Paraventricular thalamic n. (PV) | | + | | + | | −/+ | | + | |
| Paraventricular thalamic n., anterior part (PVA) | | + | | + | | −/+ | | + | |
| Paraventricular thalamic n., posterior part (PVP) | | −/+ | | + | | −/+ | | + | |
| **Hypothalamus** | |  | |  | |  | |  | |
| Medial preoptic area (MPA) | | +++ | | +++ | | ++++ | | +++ | |
| Median preoptic n. (MnPO) | | +++ | | ++++ | | +++ | | +++ | |
| Medial preoptic n., lateral part (MPOL) | | +++ | | +++ | | ++ | | ++/+++ | |
| Medial preoptic n., lateral part (MPOM) | | +++ | | +++ | | NR | | NR | |
| Ventrolateral preoptic n. (VLPO) | | ++ | | +++ | | ++ | | +/++ | |
| Ventromedial preoptic n. (VMPO) | | ++ | | + | | − | | + | |
| Strial part of the preoptic area (StA) | | −/+ | | + | | + | | − | |
| Striohypothalamic nucleus (StHy) | | ++++ | | +++ | | NR | | NR | |
| Anterior hypothalamic area, posterior part (AHP) | |  | | − | | − | | ++ | |
| Anterior hypothalamic area, anterior part (AHA) | | −/+ | | − | | − | | + | |
| Anterior hypothalamic area, central part (AHC) | | −/+ | | − | | − | | +/++ | |
| Lateral hypothalamic area (LH) | | − | | ++ | | + | | +/++ | |
| Posterior hypothalamic n. (PH) | | − | | + | | + | | + | |
| Ventral tuberomammillary n. (VTM) | | −/+ | | − | | − | | ++ | |
| Premammillary n., ventral part (PMV) | | − | | − | | + | | −/+ | |
| Retrochiasmatic Area (RCh) | | ++ | | ++ | | NR | | NR | |
| Arcuate n., dorsal part (ArcD) | | + | | ++ | | ++ | | − | |
| Arcuate n., medial part (ArcM) | | ++ | | + | | ++ | | ++ | |
| Arcuate n., lateral part (ArcL) | | ++ | | + | | + | | + | |
| Arcuate hypothalamic n., medial posterior part (ArcMP) | | +/++ | | ++ | | +++ | | +++ | |
| Arcuate hypothalamic n., lateroposterior part (ArcLP) | | +/++ | | ++ | | ++ | | ++ | |
| Paraventricular hypothalamic n., ventral part (PaV) | | ++ | | + | | ++/+++ | | +++ | |
| Paraventricular hypothalamic n., dorsal cap (PaDC) | | + | | + | | + | | + | |
| Paraventricular hypothalamic n., anterior parvicellular part (PaAP) | | + | | + | | + | | ++ | |
| Paraventricular hypothalamic n., medial parvicellular part (PaMP) | | + | | + | | ++ | | +++ | |
| Paraventricular hypothalamic n., lateral magnocellular part (PaLM) | | + | | ++ | | + | | ++ | |
| Paraventricular hypothalamic n., medial magnocellular part (PaMM) | | ++ | | + | | −/+ | | ++ | |
| Paraventricular hypothalamic n., posterior part (PaPo) | | −/+ | | +++ | | +++ | | +++ | |
| Dorsomedial hypothalamic n., dorsal part and dorsal hypothalamic  area (dDMH/DHA) | | ++ | | +++ | | +++ | | +++ | |
| Dorsomedial hypothalamic n., compact part (cDMH) | | + | | + | | + | | + | |
| Dorsomedial hypothalamic n., ventral part (vDMH) | | ++ | | +++ | | +++ | | +++ | |
| Perifornical n. (PeF) | | −/+ | | + | | − | | + | |
| Dorsal tuberomammillary n. (DTM) | | − | | − | | − | | + | |
| Medial tuberal n. (MTu) | | + | | − | | − | | + | |
| Terete hypothalamic n. (Te) | | −/+ | | − | | − | | −/+ | |
| Supraoptic n., retrochiasmatic part (SOR) | | −/+ | | − | | − | | −/+ | |
| *Brainstem* | |  | |  | |  | |  | |
| Lateral parabrachial n., ventral part (LPBV) | | +++ | | ++ | | ++ | | +++ | |
| Medial parabrachial n. (MPB) | | −/+ | | + | | − | | ++ | |
| Lateral parabrachial n., central part (LPBC) | | ++ | | +++ | | +++ | | + | |
| Lateral parabrachial n., internal part (LPBI) | | +++ | | − | | +++ | | − | |
| Lateral parabrachial n., dorsal part (LPBD) | | ++ | | − | | ++ | | − | |
| Substantia nigra (SNR) | | − | | − | | − | | − | |
| Ventral tegmental area | | − | | − | | − | | − | |
| Intermediate Reticular Nucleus (IRt) | | − | | + | | NR | | NR | |
| Pontine Reticular Nucleus (Pn) | | + | | +/− | | NR | | NR | |
| Periaqueductal Gray (PAG) | | − | | +/− | | NR | | NR | |
| Motor Trigem, tensor tymp (5TT) | | − | | + | | NR | | NR | |
| *Cerebellum* | | − | | − | | − | | − | |

^1^ Expression of tdTomato in BRS3-IRES-Cre;Ai14 mice (consensus of 3 mice, 5 months of age). Brain region nomenclature as in Franklin and Paxinos (2008), except for DMH and DHA regions. Paxinos, G. and K. B. J. Franklin (2008). The mouse brain in stereotaxic coordinates. Boston, Elsevier Academic Press.

^2^ Piñol, R. A., et al. (2018). “Brs3 neurons in the mouse dorsomedial hypothalamus regulate body temperature, energy expenditure, and heart rate, but not food intake.” Nat Neurosci 21:1530-1540.

^3^ Zhang, L., et al. (2013). "Anatomical characterization of bombesin receptor subtype-3 mRNA expression in the rodent central nervous system." J Comp Neurol 521:1020-1039.

^4^ Cell body expression (fiber staining is not included) was scored as follows: ++++, very strong expression; +++, strong expression; ++, moderate expression; +, low expression; −/+, slightly above background expression; − no expression; NR, not reported.
